# Presenting Complaints in Acute Dengue Infection and Differences in Presenting Complaints Between Primary and Secondary Dengue Infections

**DOI:** 10.7759/cureus.19320

**Published:** 2021-11-06

**Authors:** Saba Arshad, Mubariz Ahmed, Faramarz Khan, Muhammad Khurram, Basil Usman

**Affiliations:** 1 Internal Medicine, Holy Family Hospital, Rawalpindi Medical University, Rawalpindi, PAK

**Keywords:** abdominal pain, dengue shock syndrome, dengue hemorrhagic fever, dengue fever, denv, presenting complaints, secondary dengue infection, primary dengue infection

## Abstract

Aim and objectives

To describe the presenting complaints in acute dengue infection, and identify any differences in presenting complaints between primary and secondary dengue infection patients.

Material and methods

This cross-sectional observational study was conducted at the Department of Infectious Diseases and Medicine, Holy Family Hospital, Rawalpindi, from July 2019 to December 2019 during the Dengue Rawalpindi Epidemic 2019. Presenting complaints of patients who fulfilled the inclusion criteria of the study were recorded on a proforma on their admissions and their informed consent was taken. Of these patients, 70 primary and 70 secondary dengue infection patients were randomly selected for comparison of presenting complaints. The two groups were compared using the chi-square test and a P-value of <0.05 was considered significant.

Results

Intermittent fever (88.6%), headache (85%), myalgia (87.9%), arthralgia/bone pain (75%), and retro-orbital pain (47.9%) were common in most dengue patients. Hemorrhagic manifestations, such as rash (15%), epistaxis (11.4%), gum bleeding (15%), melena (7.9%), hematemesis (6.4%), hemoptysis (5.7%), and hematuria (6.4%), were less common.

Abdominal pain was significantly more common in secondary dengue infections (50% in secondary dengue infections compared to 32.9% in primary dengue infections).

Conclusions

Fever, headache, myalgia, arthralgia/bone pains, retro-orbital pain as well as rash, epistaxis, gum bleeding, melena, hematemesis, hemoptysis, hematuria, and decreased urine output despite fluid intake are presenting complaints of dengue infection.

Patients with abdominal pain in addition to the above presenting complaints are more likely to be cases of the more serious secondary dengue infection.

## Introduction

Dengue is a viral illness. It is the most frequent mosquito-borne disease in the world [1]. The principal vector is the diurnal mosquito Aedes aegypti. It can also be transmitted through non-vector means. These include needle-stick injuries, mucosal splashes, blood transfusion, and organ transplantation [2-4]. Dengue viruses (DENV) have four serotypes (DENV 1-4). These are serologically and genetically distinct [5]. DENVs are responsible for a wide spectrum of diseases ranging from a milder illness known as dengue fever (DF) to a severe disease characterized by coagulopathy, increased vascular fragility, and loss of fluid due to capillary permeability, leading to hypovolemic shock, called dengue hemorrhagic fever/dengue shock syndrome (DHF/DSS) [6].

Patients who are infected by any DENV for the first time are known as cases of primary dengue infection. Patients who were previously infected by another DENV and then present with infection from a new serotype are known as cases of secondary dengue infection.

DENV serotypes have many similar structural antigens. Upon infection with any DENV, the adaptive immune response that develops, provides long-term immunity to the homologous serotype. The antibodies induced are cross-reactive with the heterologous DENV serotypes as well and provide short-term immunity against them, lasting from anywhere between three months and two years [7-9]. After this period, infection with a heterologous serotype increases the risk for more severe infection [10], including a higher risk of developing DHF/DSS [11]. Hence, secondary dengue is considered more dangerous.

Confirmation of acute dengue is obtained by the presence of the dengue NS-1 antigen and IgM antibodies against DENV in the serum upon presentation. The presence of IgG antibodies in the serum indicates seroconversion due to prior DENV infection, hence the patient is defined as having secondary dengue infection. The absence indicates that the patient has the primary dengue infection [12-14].

Dengue infection is associated with a wide array of presenting complaints: fever, headache, and retro-orbital pain are more common symptoms. Warning signs are also described by the Center for Disease and Control Management. These signs include abdominal pain, bleeding from any site, and irritability [15].

We aimed to describe the common presenting complaints in both primary and secondary dengue infections. We also aimed to compare the possible differences in presenting complaints between the two groups. Although the presenting complaints in acute dengue infection have been well-described previously, there has been a lack of studies comparing the differences in presenting complaints between the two groups, especially in our region. Since secondary dengue infection is more lethal than primary infection, earlier suspicion and prompt management can be crucial for the prognosis. This is especially true for resource-limited settings such as ours.

## Materials and methods

This cross-sectional observational study was conducted at the Department of Infectious Diseases and Medicine, Holy Family Hospital, Rawalpindi, Pakistan, from July 2019 to December 2019, during the Dengue Rawalpindi Epidemic 2019. Ethical approval for the study was taken from the Research and Ethical Committee, Rawalpindi Medical University & Allied Hospitals, Rawalpindi (approval number R-48/RMU).

A proforma was specifically designed focusing on various symptoms of dengue infection. All patients admitted with suspected dengue infection were interviewed to fulfill the proforma at admission, after taking informed consent. Dengue IgG, IgM, and NS1 antigen testing of each patient was performed to diagnose dengue infection or otherwise, according to Dengue Expert Advisory Group (DEAG) guidelines.

Patients were categorized into primary or secondary infection based on the results of dengue IgG, IgM, and NS1 antigen. They were labeled primary dengue infection if they were positive for the NS-1 antigen and IgM against DENV. Others who also had IgG in the serum in addition to the NS-1 antigen and IgM antibodies against DENV were labeled to have secondary dengue infection. Patients were managed according to DEAG guidelines.

The total number of confirmed dengue patients was 6220. They were divided into primary and secondary dengue categories. From each category, 70 patients were included using simple randomization, i.e. 70 patients having primary dengue infection (Group-I) and 70 patients having secondary dengue infection (Group-II). The total sample size was thus 140. The presenting symptoms in both groups were collected and analyzed using statistical software (SPSS version 21). The chi-square test was used as a test of significance. A P-value of <0.05 was considered significant.

## Results

Of the 140 patients, 52% (n=74) were male and 47% (n=66) were female. In the Group-I patients, 54.2% (38) were male and 45.7% (32) were female. The mean age of the patients was 37.1 ± 5.9 years. In Group-II, 51.4% (36) were male and 48.5% (34) were female. The mean age of the patients was 33.4 ± 6.4 years. The two groups did not differ with reference to age and gender significantly (P-value > 0.05).

**Figure 1:**
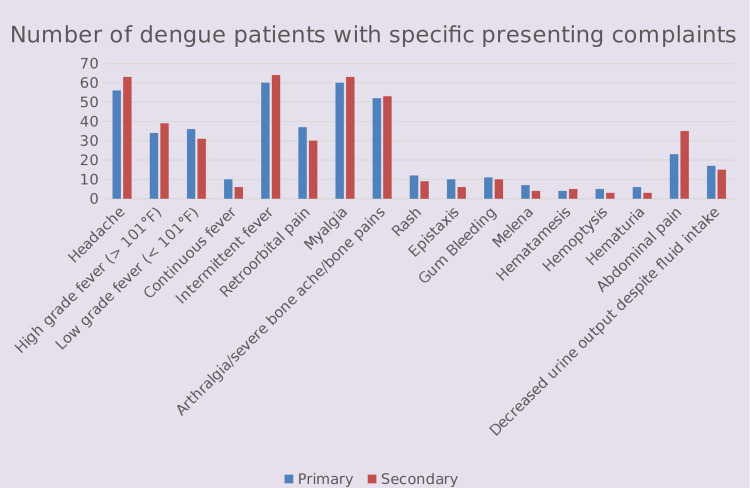
Presenting complaints in primary and secondary dengue infections

Intermittent fever, headache, myalgia, and arthralgia were the most common symptoms noted in both Group-I and Group-II patients. Melena, hemoptysis, and hematuria were the least frequently noted symptoms in all patients. Details are given in Table 1 and Figure 1.

**Table 1:** Presenting complaints in primary and secondary dengue

Presenting Complaint	Total number (n=140)	Primary Dengue (n=70)	Secondary Dengue (n=70)	P-value
Headache	119	56 (80%)	63 (90.0%)	0.0988
High-grade fever (> 101°F)	73	34 (48.6%)	39 (55.7%)	0.4021
Low-grade fever (< 101°F)	67	36 (51.4%)	31 (44.3%)	0.4021
Continuous fever	16	10 (14.3%)	6 (8.6%)	0.2913
Intermittent fever	124	60 (85.7%)	64 (91.4%)	0.2913
Retro-orbital pain	67	37 (52.8%)	30 (42.9%)	0.2427
Myalgia	123	60 (85.7%)	63 (90.0%)	0.4378
Arthralgia/severe bone ache/bone pains	105	52 (74.3%)	53 (75.7%)	0.8488
Rash	21	12 (17.1%)	9 (12.9%)	0.4881
Epistaxis	16	10 (14.3%)	6 (8.6%)	0.2913
Gum bleeding	21	11 (15.7%)	10 (14.3%)	0.8172
Melena	11	7 (10.0%)	4 (5.7%)	0.346
Hematemesis	9	4 (5.7%)	5 (7.1%)	0.736
Hemoptysis	8	5 (7.1%)	3 (4.3%)	0.4765
Hematuria	9	6 (8.6%)	3 (4.3%)	0.3021
Abdominal pain	58	23 (32.9%)	35 (50.0%)	0.0407
Decreased urine output despite fluid intake	32	17 (24.3%)	15 (21.4%)	0.6839

Low-grade fever, continuous fever, retro-orbital pain, rash, epistaxis, gum bleeding, melena, hemoptysis, hematuria, and decreased urine output despite intake were more common in Group-I. In comparison, headache, high-grade fever, intermittent fever, myalgia, arthralgia, hematemesis, and abdominal pain were more common in Group-II. We found that both the groups differed significantly with reference to abdominal pain (P-value 0.047).

## Discussion

Intermittent fever was the most frequent presenting symptom in both groups, with a total of 124 patients presenting with it. Meanwhile, only 16 patients had a continuous fever. Of the patients, 100% who presented had fever. These results are similar to a study conducted by Singh et al. who reported that fever was a presenting complaint in 100% of patients [16]. Chuang et al. also described fever in 98% of their patients [17]. However, previously, the character of the fever has not been described.

The next common presenting symptom was myalgia. A total of 60 (85.7%) patients in the primary dengue group suffered from myalgias. In the secondary dengue group, 63 (90.0%) patients complained of myalgia. Several previous studies have described myalgia as a common symptom of dengue [16-17].

Another common presenting complaint was headache. It was present in 117 of the 140 patients included in our study. Fifty-six (80.0%) of the 70 patients in the primary dengue infection group complained of headache while 63 (90.0%) of the 70 patients in the secondary group complained of headache. Headache was also described as a common presenting complaint in previous studies [16-17].

Other common presenting complaints, such as arthralgia and retro-orbital pain, as well as less common complaints like rash, epistaxis, gum bleeding, melena, hematemesis, hemoptysis, and hematuria, have also been documented in several previous studies [18-20]. However, there have been fewer studies comparing presenting complaints in primary and secondary dengue patients. One such study described that secondary dengue patients have a lower mean age and higher mean maximum temperature [21].

Abdominal pain was present in 58 of the 140 patients included in our study. Twenty-three (32.9%) of the 70 patients in the primary dengue complained of abdominal pain while 35 (50.0%) of the patients in the secondary dengue group complained of abdominal pain. This difference was significant, indicating that abdominal pain is more common in patients with secondary infected patients.

Khanna et al. previously attempted to describe the etiology of abdominal pain in dengue infection. They found the various causes in the population they studied to be acute hepatitis, acalculus cholecystitis, acute pancreatitis, appendicitis, spontaneous bacterial peritonitis, enteritis, peptic ulcer disease, and gastric erosions [22]. Secondary dengue being more severe might increase the risk of all these etiologies, which may explain the significantly higher number of patients with abdominal pain in the secondary dengue group. Several studies have described abdominal pain to be a common symptom of dengue fever [16-17]. But to our knowledge, a comparison of the incidence of abdominal pain between primary and secondary dengue patients has never been made before.

Although the fact that secondary dengue fever is more severe is well documented, there has been scant literature on the comparison of presenting complaints between primary and secondary dengue. Resource-limited settings in developing countries can get overwhelmed during epidemics such as dengue. Early identification of the more lethal secondary dengue patients by using abdominal pain as an indicator can lead to more resources being directed to them in advance. This could be a crucial factor in improving overall morbidity and mortality outcomes.

Our main limitation was the limited sample size. We propose that future work on this line should include a larger sample size so a better, more comprehensive comparison can be made.

## Conclusions

Fever, headache, myalgia, arthralgia/bone pains, retro-orbital pain, as well as rash, epistaxis, gum bleeding, melena, hematemesis, hemoptysis, hematuria, and decreased urine output despite fluid intake, are presenting complaints of dengue infection. They should alert the care providers of acute infection, especially during an epidemic.

Patients with abdominal pain in addition to the above presenting complaints are more likely to be cases of the more serious secondary dengue infection. Thus abdominal pain can be used as an early marker of secondary dengue infection. This could be crucial for prognosis, especially in resource-limited settings.
